# Disparities between IgG4-related kidney disease and extrarenal IgG4-related disease in a case–control study based on 450 patients

**DOI:** 10.1038/s41598-021-89844-7

**Published:** 2021-05-17

**Authors:** Qiaozhu Zeng, Jingyuan Gao, Xinyu Zhang, Aichun Liu, Zhenfan Wang, Ziqiao Wang, Xiying Chi, Qianyu Shi, Yi Wang, Fei Yang, Yanying Liu, Zhan-Guo Li

**Affiliations:** 1 Department of Rheumatology and Immunology, Peking University People’s Hospital, 11, Xizhimen South Street, Beijing, 100044 China; 2 Department of Rheumatology and Immunology, Affiliated Hospital of North China University of Science and Technology, Tangshan, 06300 Hebei China; 3 Department of Radiology, Peking University People’s Hospital, Beijing, 100044 China; 4 Department of Nephrology, Peking University People’s Hospital, Beijing, 100044 China; 5 Department of Pathology, Peking University People’s Hospital, Beijing, 100044 China

**Keywords:** Immunology, Autoimmunity, Rheumatology, Rheumatic diseases

## Abstract

We aimed to compare the demographic, clinical and laboratory characteristics between IgG4-related kidney disease (IgG4-RKD+) and extrarenal IgG4-related disease (IgG4-RKD−) in a large Chinese cohort, as well as describing the radiological and pathological features of IgG4-RKD+. We retrospectively analyzed the medical records of 470 IgG4-related disease (IgG4-RD) patients at Peking University People’s Hospital from January 2004 to January 2020. The demographic, clinical, laboratory, radiological and pathological characteristics between IgG4-RKD+ and IgG4-RKD− were compared. Twenty IgG4-RD patients who had definite etiology of renal impairment including diabetes, hypertension and etc. were excluded. Among the remained 450 IgG4-RD patients, 53 were diagnosed with IgG4-RKD+ . IgG4-RKD+ patients had older age at onset and at diagnosis. Male to female ratio of IgG4-RKD+ patients is significantly higher. In the IgG4-RKD+ group, the most commonly involved organs were salivary gland, lymph nodes and pancreas. It was found that renal function was impaired in approximately 40% of IgG4-RKD+ patients. The most common imaging finding is multiple, often bilateral, hypodense lesions. Male sex, more than three organs involved, and low serum C3 level were risk factors for IgG4-RKD+ in IgG4-RD patients. These findings indicate potential differences in pathogenesis of these two phenotypes.

## Introduction

IgG4-related disease (IgG4-RD) is a systemic fibroinflammatory immune-mediated disorder, which is characterized by high levels of serum IgG4, dense lymphoplasmacytic infiltration with IgG4-positive plasma cells in multiple organs, featured storiform fibrosis, and obliterative phlebitis^1–4^.

In 2001, IgG4-RD was first described in pancreas, which was called type 1 autoimmune pancreatitis (AIP)^5^. Subsequent studies have revealed that IgG4-RD can affect any organ system. IgG4-related kidney disease (IgG4-RKD+) refers to the renal lesions in association with IgG4-RD. Previous studies have reported the kidney involvement in approximately 12–23% of total IgG4-RD patients^4,6–8^. IgG4-RKD+ could be manifested by a variety of clinical and histological characteristics, such as tubulointerstitial nephritis (TIN), membranous glomerulonephropathy (MGN) and other infrequent glomerular lesions, pyelitis, hydronephrosis and acute renal failure^9,10^.

To minimize the impact of irreversible renal damage resulting from the disease itself or unnecessary surgical intervention, early recognition and treatment are of pivotal importance. In this study, demographic, clinical, and laboratory disparities in the 53 IgG4-RKD+ and 397 IgG4RKD− patients were investigated. To our best knowledge, this is the largest study to compare the two phenotypes of patients with or without IgG4-RKD+. Determining the characteristics of these two phenotypes may help identify risk factors and their potential differences in pathogenesis.

## Methods

### Patients

**Figure 1:**
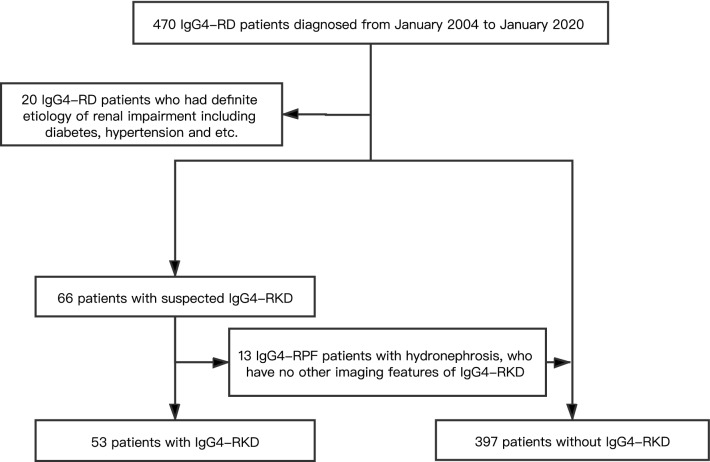
Flowchart of study design.

From January 2004 to January 2020, four hundred and seventy IgG4-RD patients presented at Peking University People's Hospital with complete clinical data. Patients were screened for IgG4-RKD+ in case of unexplained urinary abnormalities, renal imaging, or histology suggestive of IgG4 RKD+ or if they had renal involvement with conditions known to be associated with IgG4-RD. Patients who had any definite etiology of renal impairment were excluded from the study^11,12^. Twenty IgG4-RD patients who had definite etiology of renal impairment including diabetes, hypertension and etc. were excluded. Therefore, four hundred and fifty IgG4-RD patients were enrolled into the cohort (Fig. 1). Based on the 2011 comprehensive IgG4-RD diagnostic criteria^9^, there were 216 (48.0%), 35 (7.8%) and 199 (44.2%) cases diagnosed with definite, probable and possible IgG4-RD, respectively (Table 1).

**Table 1:** Demographic, clinical features and laboratory findings of 450 IgG4-RD patients.

Characteristics	All (n = 450)	IgG4-RKD+ (n = 53)	IgG4-RKD− (n = 397)	*P *value
**Demographics**				
Age at disease onset, years, median (IQR)	55 (46,62)	58 (48, 68)	54 (46, 61)	0.048**
Age at diagnosis, years, median (IQR)	57 (49,64)	60 (54, 71)	56 (48, 63)	0.013**
Time from onset to diagnosis, years, median (IQR)	1 (0,3)	1 (0, 4)	1 (0, 3)	0.357
Follow-up period, months, median (IQR)	29.8 (10.9, 52.6)	32.0 (15.4, 60.0)	28.6 (10.5, 52.6)	0.229
Disease duration, months, median (IQR)	52.7 (27.9, 85.7)	66.6 (29.8, 104.4)	52.1 (27.0, 83.1)	0.188
Gender (Male: Female)	1.4:1	2.8:1	1.2:1	0.012**
**Clinical features** **n** (%)				
Complication of allergic disease	185 (41.1)	18 (34.0)	167 (42.1)	0.260
Number of involved Organs ≥ 3	293 (65.1)	47 (88.7)	246 (62.0)	< 0.001**
Lymph node	205 (45.6)	27 (50.9)	178 (44.8)	0.402
Paranasal sinus	61 (13.6)	10 (18.9)	51 (12.9)	0.229
Thyroid gland	46 (10.2)	7 (13.2)	38 (9.8)	0.445
Lung	99 (25.8)	21 (35.9)	99 (25.8)	0.122
Liver	10 (2.2)	3 (5.7)	7 (1.8)	0.071
Pancreas	150 (33.3)	25 (47.2)	125 (31.5)	0.023**
Biliary system	76 (16.9)	12 (22.6)	64 (16.1)	0.234
Gallbladder	59 (13.1)	12 (22.6)	47 (11.8)	0.029**
Retroperitoneal fibrosis	66 (14.7)	11 (20.8)	55 (13.9)	0.182
Mesentery	5 (1.1)	1 (1.9)	4 (1.0)	0.566
Aorta	15 (3.3)	3 (5.7)	11 (3.0)	0.315
Prostate	20 (4.4)	5 (9.4)	15 (3.8)	0.061
Salivary gland	295 (65.6)	32 (60.4)	263 (66.3)	0.398
Lacrimal gland	218 (48.4)	22 (41.5)	196 (49.4)	0.282
Parotid gland	152 (33.8)	17 (32.1)	135 (34.0)	0.780
**Laboratory findings**				
Serum IgG4 (mg/dL), median (IQR)	1030 (348, 1660)	1300 (359, 2355)	1000 (348, 1590)	0.163
Serum IgE (IU/ml), median (IQR)	286.5 (103.4, 742.6)	288.2 (171.1, 596.8)	286.5 (103.0, 761.7)	0.594
CRP (mg/dl), median (IQR)	2.6 (1.2, 8.2)	2.6 (1.0, 8.9)	2.6 (1.2, 8.1)	0.659
ESR (mm/h), median (IQR)	17 (8, 45)	20 (10, 69)	16 (8, 40)	0.097
Eosinophil count (10^9^/L), median (IQR)	0.2 (0.1, 0.4)	0.2 (0.1, 0.5)	0.2 (0.1, 0.4)	0.248
C3 (g/L), mean (SD)	0.9 ± 0.4	0.7 ± 0.4	0.9 ± 0.3	0.007**
C4 (g/L), median (IQR)	0.2 (0.1,0.3)	0.2 (0.1,0.2)	0.2 (0.2,0.3)	0.167
Hb(g/L), median (IQR)	134 (121, 145)	125 (110, 142)	135 (123, 145)	0.032**

IgG4-RKD + vs IgG4-RKD −

**P < 0.05.

Renal pathological findings were available for 6 patients: dense lymphoplasmacytic infiltration with more than 10 infiltrating IgG4-positive plasma cells per high-power field and an IgG4/IgG plasma cell ratio of more than 40% with fibrosis. In total, there were 53 patients diagnosed with IgG4-RKD+ based on the diagnostic criteria of IgG4-RKD+ proposed in 2011^13^. All the kidney biopsy were reviewed by a pathologist.

Gender, age, allergic diseases, clinical manifestations, organ involvement, radiological and pathological findings were recorded. According to the criteria from the European Academy of Allergy and Clinical Immunology (EAACI), allergic disease was diagnosed by experienced specialists. This study was approved by the Medical Ethics Committee of Peking University People’s Hospital (Beijing, China). According to national regulations and in accordance with the Declaration of Helsinki, data protection authority and medical research ethical committee gave their approval.

### Laboratory features, imaging examination and histological studies

We recorded laboratory tests, including serum IgG4 level, serum IgE level, C-reactive protein (CRP), erythrocyte sedimentation rate (ESR), eosinophilia, serum C3, serum C4, hemoglobulin (Hb), and renal function (including urine specific gravity, PH, proteinuria, urinary white blood cells, urine occult blood, urea nitrogen, creatinine, estimated glomerular filtration rate (eGFR), serum β2-microglobulin (β2-MG), urine N-acetyl-β-D-glucosidase (NAG), and retinol-binding protein (RBP)).

All patients underwent radiology examinations consisting of Computed Tomography (CT), or Magnetic resonance imaging (MRI), and some patients also received 18F-fluorodeoxyglucose PET-CT.

We fixed all tissue biopsy samples in formalin and embedded in paraffin, then stained them with hematoxylin and eosin (H&E) and immunocytochemistry (IHC). IHC was performed using the avidin–biotin complex-peroxidase method with monoclonal antibody to human IgG4 (Zymed, Carlsbad, CA; dilution 1:50 or 1:100 depending on staining laboratory) on sections from paraffin-embedded tissue.

The renal biopsy was examined specifically for features suggestive of IgG4-RKD+, including presence of TIN, lymphoplasmacytic infiltration, as well as tubulointerstitial presentations on light microscopy, glomerular light microscopy abnormalities, glomerular or tubular basement membrane (TBM) deposits on IF or EM. IgG4 staining was done and the number of positive plasma cells/hpf were calculated.

### Statistical analysis

Using Stata software (V.15.0; Stata, College Station, TX, USA), data were analyzed by descriptive methods, with standard summary statistics including mean (S.D.), median, interquartile range (IQR), and proportions. We performed the Student’s *t* test for differences for continuous, normally distributed data; continuous, non-normally distributed data were analyzed by the Mann–Whitney test. Categorical variables were processed by *X*^2^ or Fisher’s exact tests. Logistic regression analysis with enter method was performed to compare the IgG4-RKD+ and IgG4-RKD− patients. Factors with *P* < 0.05 and clinical significance in the univariate analysis were included in the multivariate analysis. *P* < 0.05 was considered to be statistically significant.

## Results

### Demographic features

Demographic details of all the 450 IgG4-RD patients were outlined in Table 1. Among the 450 patients, there were 53 (11.8%) patients with IgG4-RKD+, and 397 (88.2%) patients with IgG4-RKD−. The comparison of demographic characteristics of IgG4-RKD+ and IgG4-RKD− patients was also indicated in Table 1. Compared with IgG4-RKD− patients, IgG4-RKD+ patients had older age at onset and older age at diagnosis (58 vs 54, *P* = 0.048; 60 vs 56, *P* = 0.013, respectively). Male to female ratio of IgG4-RKD+ patients is significantly higher (2.8:1 vs 1.2:1, *P* = 0.012).

### Clinical characteristics

**Figure 2:**
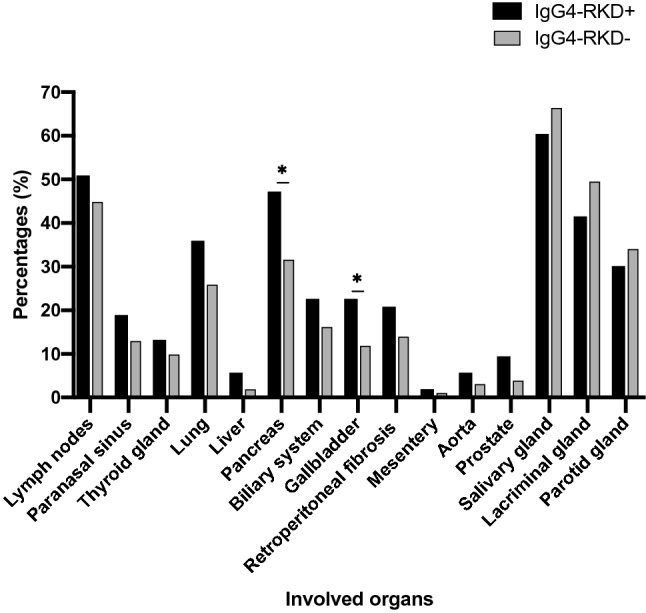
Summary of affected organs in IgG4-RKD+ and IgG4-RKD− patients at baseline.

As shown in Table 1, there were more patients with involved organs of 3 or more in the IgG4-RKD + group than that in the IgG4-RKD− group (88.7% vs 62.0%, *P* < 0.001). Detailed organ involvement between IgG4-RKD + and IgG4-RKD− groups was shown in Fig. 2. In the IgG4-RKD+ group, the most commonly involved organs were salivary gland (60.4%), lymph nodes (50.9%) and pancreas (47.2%). While in the IgG4-RKD− group, salivary gland (66.3%) was the most commonly affected organ, followed by lacrimal gland (49.4%), and lymph nodes (44.8%). The involvement of pancreas (47.2% vs 31.5%, *P* = 0.023) and gallbladder (22.6% vs 11.8%, *P* = 0.029) were more common in IgG4-RKD+ patients.

### Laboratory parameters

Disparities in laboratory features between the IgG4-RKD+ and IgG4-RKD− groups were indicated in Table 1. No significant differences were found in levels of serum CRP, IgG4 and IgE, eosinophil count, ESR and serum C4. Serum C3 (0.7 ± 0.4 vs 0.9 ± 0.3, *P* = 0.007) and Hb (125 vs 135, *P* = 0.032) were significantly lower in the IgG4-RKD+ group.

### Renal damage indexes of the IgG4-RKD+ group

As presented in Table 2, it was found that renal function was impaired with the reduced eGFR (37.7%), elevated blood urea nitrogen (32.1%) and elevated serum creatine (22.7%). The median eGFR was 84.9 mL/min/1.73 m^2^ (IQR, 71.5, 101.2). The level of serum creatinine varied from 61 to 98µmol/L at baseline. The median blood urea nitrogen was 6.7 mmol/L (IQR, 4.4, 8.5). Fourteen (26.4%) of the 53 IgG4-RKD+ patients presented with proteinuria (1+ or greater on dipstick). Approximately 40% of IgG4-RKD+ patients presented with urine occult blood. Positive urinary white blood cells (urinary white blood cells > 14/µl) excluding urinary infection were detected in 10 (18.9%) patients.

**Table 2:** Renal indexes in IgG4-RKD+ patients (n = 53).

Characteristics	Values
Urine specific gravity, median (IQR)	1.011 (1.009, 1.020)
Reduced gravity, n (%)	3 (5.7)
PH, median (IQR)	6 (5.5, 6.5)
Reduced PH, n (%)	0 (0)
Proteinuria (1+ or greater on dipstick), n (%)	14 (26.4)
Urine occult blood (+), n (%)	21 (39.6)
Urinary white blood cells (+), n (%)	10 (18.9)
Estimated GFR, mL/min/1.73 m^2^	84.9 (71.5, 101.2)
Reduced estimated GFR (ml/min/1.73m2), n (%)	20 (37.7)
Blood urea nitrogen (mmol/L), median (IQR)	6.7 (4.4, 8.5)
Elevated blood urea nitrogen (mmol/L), n (%)	17 (32.1)
Serum creatine (umol/L), median (IQR)	82.5 (61, 98)
Elevated serum creatine (umol/L), n (%)	12 (22.7)
Renal tubular function test*	
β2-MG (µg/L), median (IQR)	3172.1 (685.2, 8167.2)
Elevated β2-MG (µg/L), n (%)	11 (91.7)
Urine N-acetyl-β-D-glucosidase(NAG) (U/L), median (IQR)	21.1 (14.5, 37.3)
Elevated urine N-acetyl-β-D-glucosidase(NAG) (U/L), n (%)	11 (91.7)
Retinol-Binding Protein (RBP) (mg/L), median (IQR)	1.2 (0.6, 5.9)
Elevated retinol-Binding Protein (RBP) (mg/L), n (%)	8 (66.7)

*Twelve (22.6%) of the 53 IgG4-RKD+ patients had been tested for renal tubular function.

The renal tubular function was also impaired. Urine specific gravity was reduced in 5.7% of the 53 patients. Among the 12 IgG4-RKD+ patients who had specific renal tubular function tested, the three indexes of β2-MG, NAG and RBP were simultaneously elevated in 6 (50%) patients. The median β2-MG, NAG and RBP of all the IgG4-RKD+ patients were 3172.1 µg/L (IQR, 685.2, 8167.2), 21.1 U/L (IQR, 14.5, 37.3) and 1.2 mg/L (IQR, 0.6, 5.9), respectively, which were all higher than the normal range.

### Risk factors associated with IgG4-RKD+

As shown in Table 3, older age at diagnosis (OR = 1.030, 95% CI, 1.005–1.056; *P* = 0.020), ≥ 3 organs involvement (OR = 4.808, 95% CI, 2.007–11.517; *P* < 0.001), pancreas (OR = 1.943, 95% CI, 1.088–3.468; *P* = 0.025), gallbladder (OR = 2.179, 95% CI, 1.070–4.441; *P* = 0.032), , high level of ESR (OR = 1.010, 95% CI, 1.000–1.020; *P* = 0.044) were associated with higher rate of IgG4-RKD + in univariate analysis. Female (OR = 0.446, 95% CI, 0.235–0.848; *P* = 0.014), serum C3 levels (OR = 0.270, 95% CI, 0.106–0.687; *P* = 0.006) and Hb (OR = 0.980, 95% CI, 0.965–0.996; *P* = 0.014) were the protective factors of IgG4-RKD + .

**Table 3:** Univariate analysis of logistic regression of risk factors for IgG4-RKD+ patients.

Characteristics	Univariate analysis		
	*P*-value	OR	95%CI
**Demographics**			
Age at disease onset, median (IQR)	0.072	1.022	0.998, 1.047
Age at diagnosis, median (IQR)	0.020**	1.030	1.005, 1.056
Time from onset to diagnosis, median (IQR)	0.930	1.003	0.931, 1.078
Follow-up period, months, median (IQR)	0.802	1.001	0.932, 1.080
Disease duration, months, median (IQR)	0.611	1.001	0.996, 1.006
Female, n (%)	0.014**	0.446	0.235, 0.848
**Clinical features**			
Complication of allergic disease, n (%)	0.262	0.708	0.388, 1.294
**Number of involved organs, n (%)**			
1–2 organs	Ref	Ref	Ref
≥ 3 organs	< 0.001**	4.808	2.007, 11.517
Lymph node	0.403	1.278	0.720, 2.268
Paranasal sinus	0.232	1.578	0.747, 3.334
Thyroid gland	0.447	1.397	0.590, 3.305
Lung	0.110	1.638	0.894, 2.999
Liver	0.087	3.343	0.837, 13.343
Pancreas	0.025**	1.943	1.088, 3.468
Biliary system	0.237	1.523	0.759, 3.057
Gallbladder	0.032**	2.179	1.070, 4.441
Retroperitoneal fibrosis	0.186	1.629	0.791, 3.354
Mesentery	0.573	1.889	0.207, 17.229
Aorta	0.323	1.925	0.525, 7.056
Prostate	0.070	2.653	0.923, 7.624
Salivary gland	0.399	0.776	0.431, 1.398
Lacrimal gland	0.284	0.728	0.407, 1.301
Parotid gland	0.780	0.916	0.496, 1.692
**Laboratory findings**			
Serum IgG4 (mg/dL), median (IQR)	0.293	1.000	1.000, 1.000
Serum IgE (IU/ml), median (IQR)	0.924	1.000	0.999, 1.001
CRP (mg/dl), median (IQR)	0.516	0.993	0.973, 1.014
ESR (mm/h), median (IQR)	0.044**	1.010	1.000, 1.020
Eosinophil count (109/L), median (IQR)	0.072	1.526	0.963,2.418
C3(g/L), mean (SD)	0.006**	0.270	0.106,0.687
C4(g/L), median (IQR)	0.446	0.442	0.054, 3.608
Hb(g/L), median (IQR)	0.039**	0.984	0.969, 0.999

**P < 0.05.

**Figure 3:**
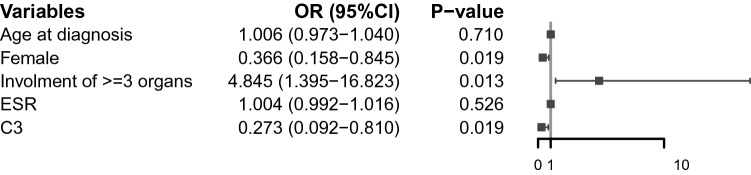
Multivariate analysis of logistic regression.

However, only female (OR = 0.366, 95% CI, 0.158–0.845; *P* = 0.019), ≥ 3 organs involvement (OR = 4.845, 95% CI, 1.395–16.823; *P* = 0.013), and serum C3 levels (OR = 0.273, 95% CI, 0.092–0.810; *P* = 0.019) remained significant after multivariate analysis (Fig. 3).

### Pathological findings

In total, 6 patients with IgG4-RKD+ underwent renal biopsy; all of the 6 (100%) patients had TIN. TIN in 1 case (16.6%) was associated with glomerular disease. Membranous nephropathy was the cause of glomerular disease in this case.

All the patients with TIN had a lymphoplasmocytic (LPC) infiltrate with fibrosis. The LPC infiltration was diffuse in 3 patients and patchy in the other 3 patients. In all of the 6 patients with TIN, IgG4 staining demonstrated > 10 IgG4+ plasma cells per high-power field (in the most concentrated area), and all these patients fulfilled the Raissian criteria for IgG4-TIN^14^.

### Radiological findings

**Figure 4:**
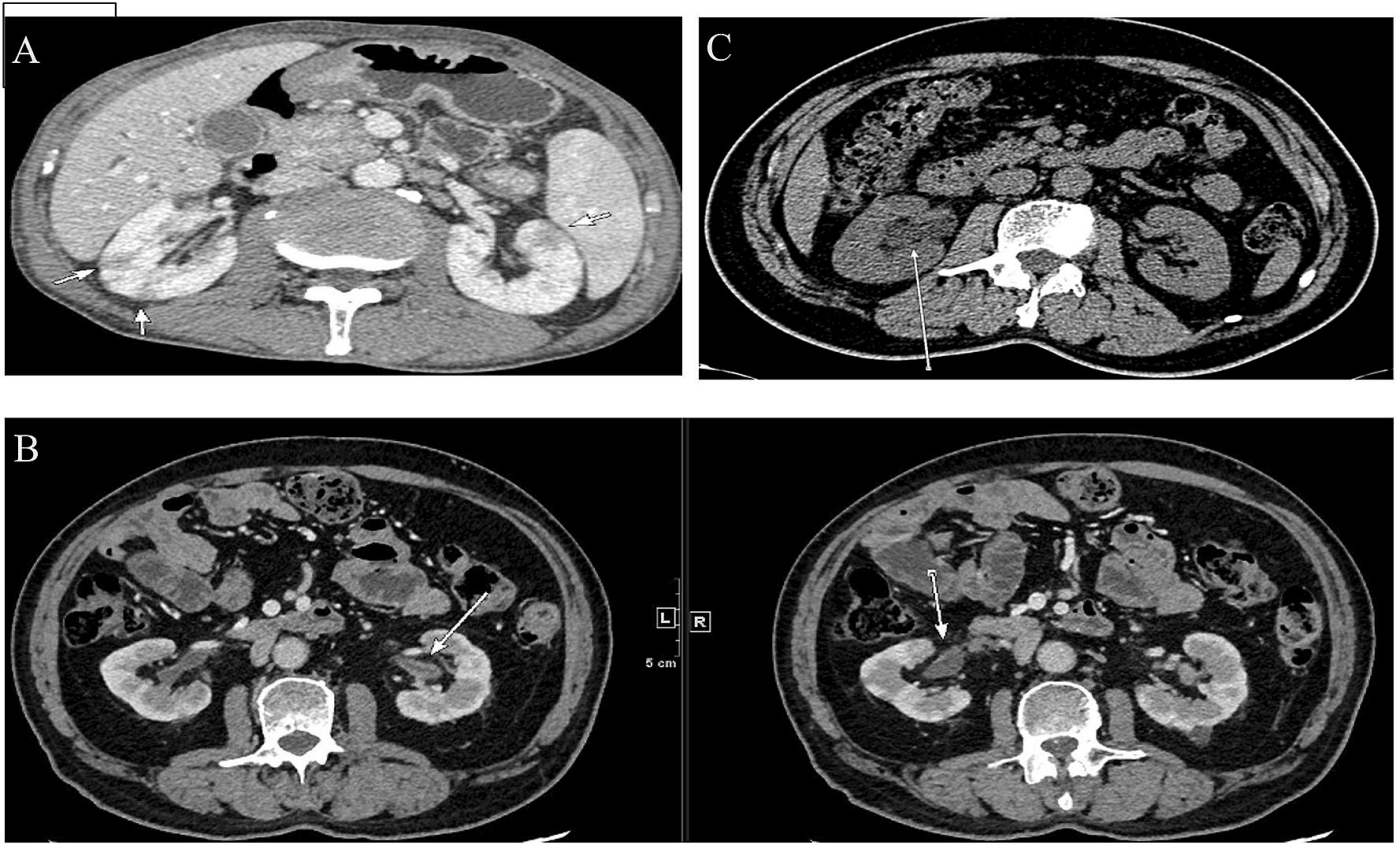
Representative contrast-enhanced CT imaging of IgG4-RKD+ . (**A**) Multiple low-density lesions in the bilateral kidneys (arrows). (**B**) ureteric obstruction and hydronephrosis related to RPF (arrow). (**C**) Renal pelvis thickening with smooth intraluminal surface (arrows).

We used contrast-enhanced CT to identify radiological abnormalities in IgG4-RKD+ patients except those with renal dysfunction. Fifty of the 53 IgG4-RKD+ patients exhibited characteristic findings of the kidney radiology. Among them, 14 patients were presented with more than one kind of lesion. The most common finding is multiple, often bilateral, hypodense lesions in 31 (58.5%) IgG4-RKD+ patients, which are called small cortical hypodense nodules (Fig. 4A), followed by thickening of the renal pelvic wall in 18 (34.0%) IgG4-RKD+ patients (Fig. 4B), and ureteric obstruction and hydronephrosis related to RPF in 9 (17.0%) patients who had also other specific kidney lesions of IgG4-RKD+ (Fig. 4C). Besides, diffuse patchy involvement, tumor-like less-enhanced mass and rim-like lesion were observed in 8 (15.1%), 2 (3.8%) and 1(1.9%) patient, respectively.

## Discussion

In this study, we compared the demographic, clinical, and laboratory characteristics of 53 IgG4-RKD+ patients and 397 IgG4-RKD− patients, as well as describing the radiological and pathological findings in patients with IgG4-RKD+. To our best knowledge, this is the largest case–control study of IgG4-RKD+ and IgG4-RKD− phenotypes.

IgG4-RD is manifested by typical clinical features, including tumor-like lesions, dense infiltration with IgG4-positive plasma cells, and extensive fibrosis of multiple organs. There is a great variability of disease manifestations for IgG4-RD, and the identification of different IgG4-RD subgroups is crucial, as a consequence of significant disparities in the characteristics of IgG4-RD regarding different organs^6,15^. In our study, the frequency of kidney involvement in IgG4-RD patients was 11.8%, which is lower than that of Japan (23.7%) and Mexico (24.6%)^6,16^, but similar to that of UK study^11^. The heterogeneity of IgG4-RKD+ definition and ethnicity in various studies may explain for this diversity. The following organs were found more commonly involved in IgG4-RKD+ patients, including salivary gland, lymph nodes and pancreas. In addition, multi-organ involvement was common in IgG4-RKD+ patients. Therefore, it is necessary to perform a general checkup to obtain a comprehensive view of the patients especially for patients with IgG4-RKD+.

Male sex, involvement of three or more organs, and low serum C3 level were risk factors for IgG4-RKD+ in IgG4-RD patients. IgG4-RD is a multi-organ immune-mediated condition that could influence almost any organ system in the body. More involved organs may suggest the higher activity of disease. In most cases, IgG4-RKD+ is diagnosed in the context of known extrarenal IgG4-RD or active status of IgG4-RD. With progressive renal decline or detection of characteristic radiological features when evaluating extrarenal IgG4-RD, kidney involvement became evident^14,17^, which may explain for the association between involvement of 3 or more organs and IgG4-RKD+. Renal involvement may appear as an intrinsic kidney disease (IgG4-RKD+) or as a consequence of ureteric obstruction from retroperitoneal fibrosis (IgG4-RPF). IgG4-RPF often concentrated in the periaortic region and ureters can be entrapped, leading to hydronephrosis and renal injury. Therefore, it is necessary to distinguish between the kidney lesions from IgG4-RKD+ and IgG4-RPF. Similar to previous studies^18^, male predominance in IgG4-RKD+ may be explained by the viewpoint that female patients were more likely to present with superficial organ involvement, while male patients with internal organ involvement.

There are some interpretations for the association of low serum C3 levels and IgG4-RKD+. The first descriptions of IgG4 TIN was previously described as “idiopathic hypocomplementemic tubulointerstitial nephritis” with extensive tubulointerstitial deposits^19^. Only about 16–34% of IgG4-RD patients have low serum complement levels, despite that hypocomplementemia is a feature of IgG4-RD. Nevertheless, more than 50% of patients with active IgG4-TIN have low concentration of complement^14,17^. Therefore, hypocomplementemia is considered an important serological feature of the disease^13^. In our study, hypocomplemenemia was found in 32 (60.4%) of IgG4-RKD+ patients, which is similar to previous studies. Wang Rong et al. found that complement activation may promote the development of IgG4-RKD+^20^. Therefore, this study added insights into the hypocomplementemia in IgG4-RKD+ patients. Low serum C3 level was found a risk factor for the development of IgG4-RKD+. Hypocomplementaemia is not characteristic feature of most IgG4-RD patients, which often suggests the existence of IgG4-RKD+, thus scrutiny is necessary.

In previously reported studies, kidney function in IgG4-RKD+ patients varies from normal to renal failure, and the development of renal dysfunction also varies from relatively acute to slowly progressive^13,14,16,17,21^. In our cohort, the renal function was impaired manifesting as the reduced eGFR, elevated blood urea nitrogen, elevated serum creatine and abnormal specific renal tubular function test. It could be attributed to IgG4-related TIN in the patients, or to the glomerular disease. IgG4-related TIN occurred in the vast majority of IgG4-RKD+ patients, and MGN was reported less than that. Consistent with the rates in previous studies, IgG4-related TIN occurred in all the 6 IgG4-RKD+ patients who had received renal biopsy. In the urinalysis in IgG4-related TIN, we could find typically mild to moderate proteinuria, as well as occasionally the presence of white blood cells^17^, which was also accordant with our result. For IgG4-RD patients, it is necessary to carry out urine routine test and renal function test (both glomerular and tubular function), in order to timely detect glomerular and renal tubular lesions.

Main abnormalities on renal imaging were revealed in a total of 42 (79.2%) of IgG4-RKD+ patients: multiple low-density nodules, hydronephrosis and thickening of renal pelvic wall. Similar with previous studies, there were also some other imaging manifestations in our cohort, including diffuse patchy involvement of the bilateral kidneys and rim-like lesion of the kidney^11,22^. CT was the most common mode of renal imaging, including PET-CT, which is increasingly used. PET-CT could contribute to excluding malignancy with little radiative damage. Moreover, PET-CT is helpful for discovering the involvement of some silent lesions, however, its cost should also be taken into account.

One of the limitations of this study is its retrospective nature, indicating that some affected organs may be neglected, although most patients have undergone general examinations, including FDG-PET. Moreover, only a small number of IgG4-RKD+ cases were diagnosed based on renal biopsy. Compared to biopsy from superficial tissue, there would be higher risk of iatrogenic trauma when patients receive deep kidney biopsy, thus some patients would not accept it. In addition, although our study has the relatively largest sample size yet, the number of patients is still small. We should cautiously interpret the results.

## Conclusion

In summary, we have specified demographic, clinical, and laboratory differences between IgG4-RKD+ patients and IgG4-RKD− patients. IgG4-RKD+ patients had older age at onset and older age at diagnosis. The male to female ratio of IgG4-RKD+ patients is significantly higher. The most commonly involved organs of IgG4-RKD+ patients were salivary gland, lymph nodes and pancreas. Male sex, involvement of three or more organs, and low serum C3 level were risk factors for IgG4-RKD+ in IgG4-RD patients. These findings indicate potential differences in pathogenesis of these two phenotypes with or without kidney involvement.

## Data Availability

The datasets generated and/or analyzed during the current study are not publicly available for ethical reasons, as well as privacy reasons but are available from the corresponding author on reasonable request.
